# Choral Voices, Communal Vulnerability

**DOI:** 10.3201/eid3211.AC3211

**Published:** 2026-11

**Authors:** Terence Chorba

**Affiliations:** Centers for Disease Control and Prevention, Atlanta, Georgia, USA

**Keywords:** Edgar Degas, Les Choristes, respiratory infections, art–science connection

**Figure 1:**
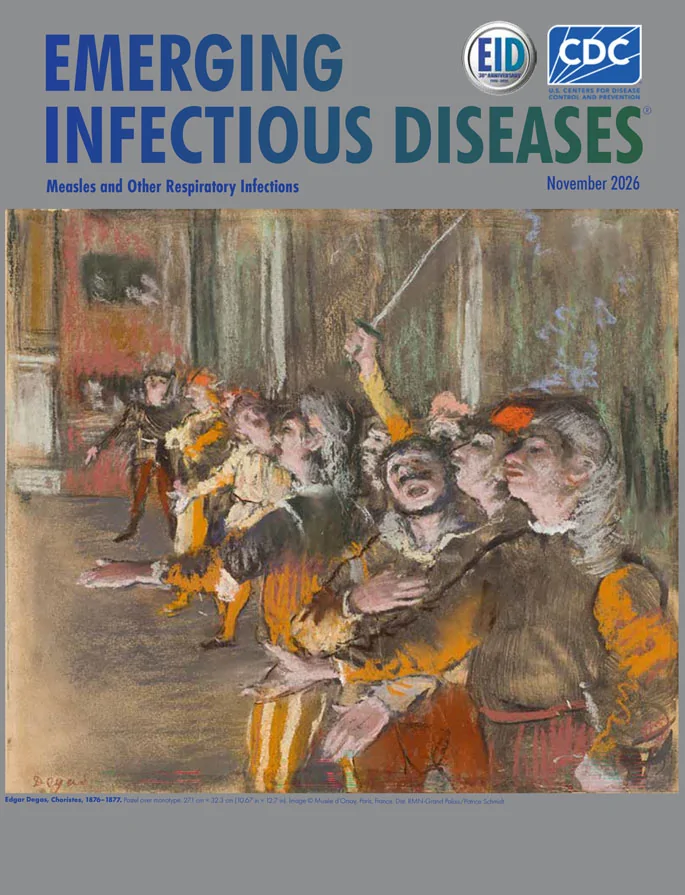
**Edgar Degas (1834–1917), *Les Choristes*,** 1877. Pastel on monotype. 32 cm × 27 cm (10.6 in × 12.6 in). Image © Musée d’Orsay, Paris, France. Dist. RMN-Grand Palais/Patrice Schmidt.

On the cover of this month’s issue, the oil and pastel artwork *Les Choristes* portrays a chorus of singers on stage, captured during an animated, operatic performance. The singers stand in proximity, sharing airspace as they project their voices. The creator of *Les Choristes*, Edgar Degas (1834–1917), was a pivotal figure in Impressionism, known for his depictions of ballet dancers, opera performers, and scenes of modern Parisian life. He was born in Paris into a well-to-do family, schooled in a prestigious lycée, and enrolled in the Faculty of Law at the Sorbonne. However, by age 21, he had demonstrated considerable artistic talent with technical drawings and enrolled in the École des Beaux-Arts in Paris with an intent to become a realist painter of historical scenes and settings.

Working across multiple media, including pastel, oil painting, sculpture, and printmaking, Degas eventually became fascinated by modern performance artists and the spaces in which they practiced and performed. Degas’ *Les Choristes* vividly reflects his interest in the theater, an environment that is often associated with intense physical exertion, close human contact, and exposure to other conditions that are conducive to the transmission of respiratory infection.

Completed in 1877, *Les Choristes* also offers insight into Degas’ artistic development and his innovative approach to representing movement and experimenting with the human form. By the mid-1870s, he had shifted his attention from historical subjects to scenes of contemporary life, especially those involving music and dance. Opera houses and ballet theaters became recurring subjects in his work, mirroring both his personal interests and the vibrant cultural life of the *Belle Époque*, the interwar years of 1871 through 1914, when Paris was renowned for cultural innovation and tremendous urban development. Although more than half of Degas’ other works depict dancers, several feature solo singers either performing or rehearsing (Figures 1, 2).

**Figure 2:**
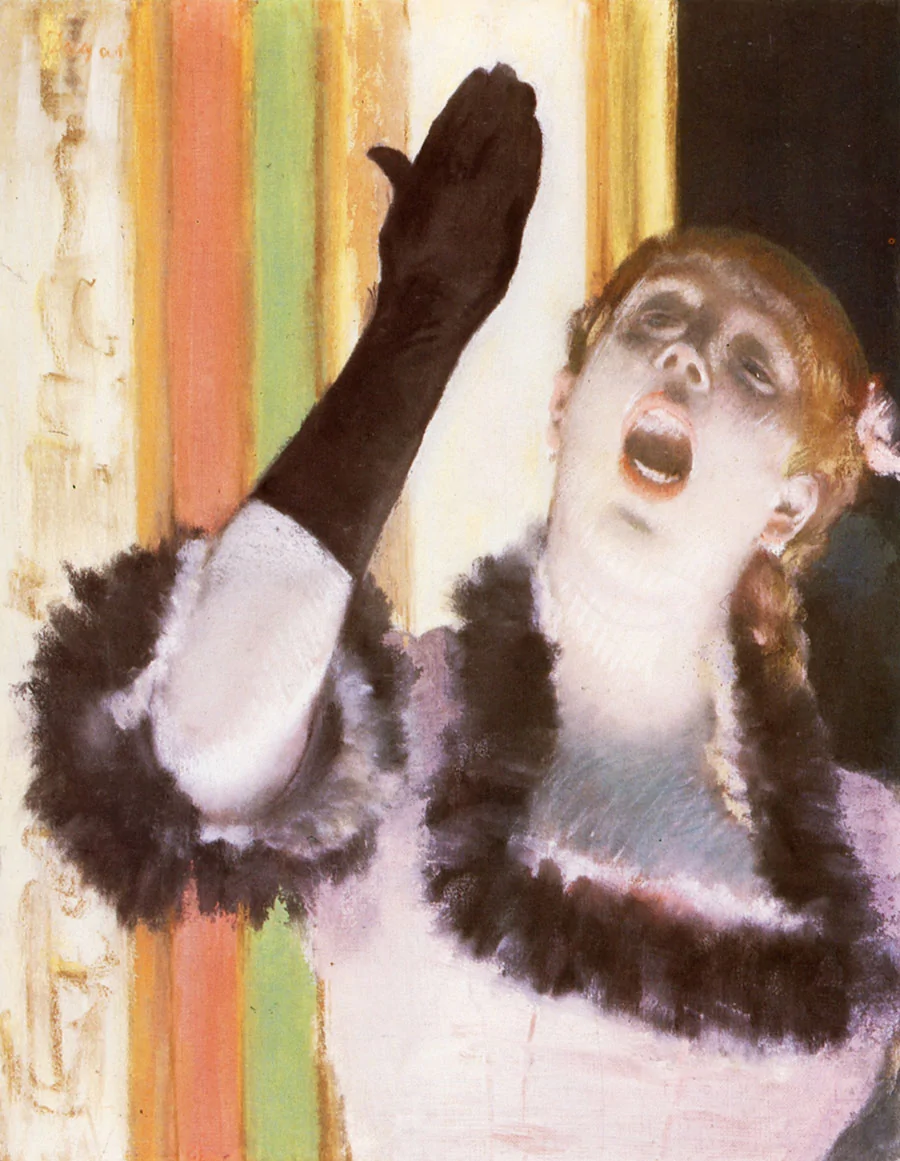
Edgar Degas, *Singer with a Glove*, 1878. Pastel on canvas. 53.2 × 41 cm (20.938 in × 16.125 in). Fogg Museum, Harvard University, Cambridge, Massachusetts, USA.

**Figure 3:**
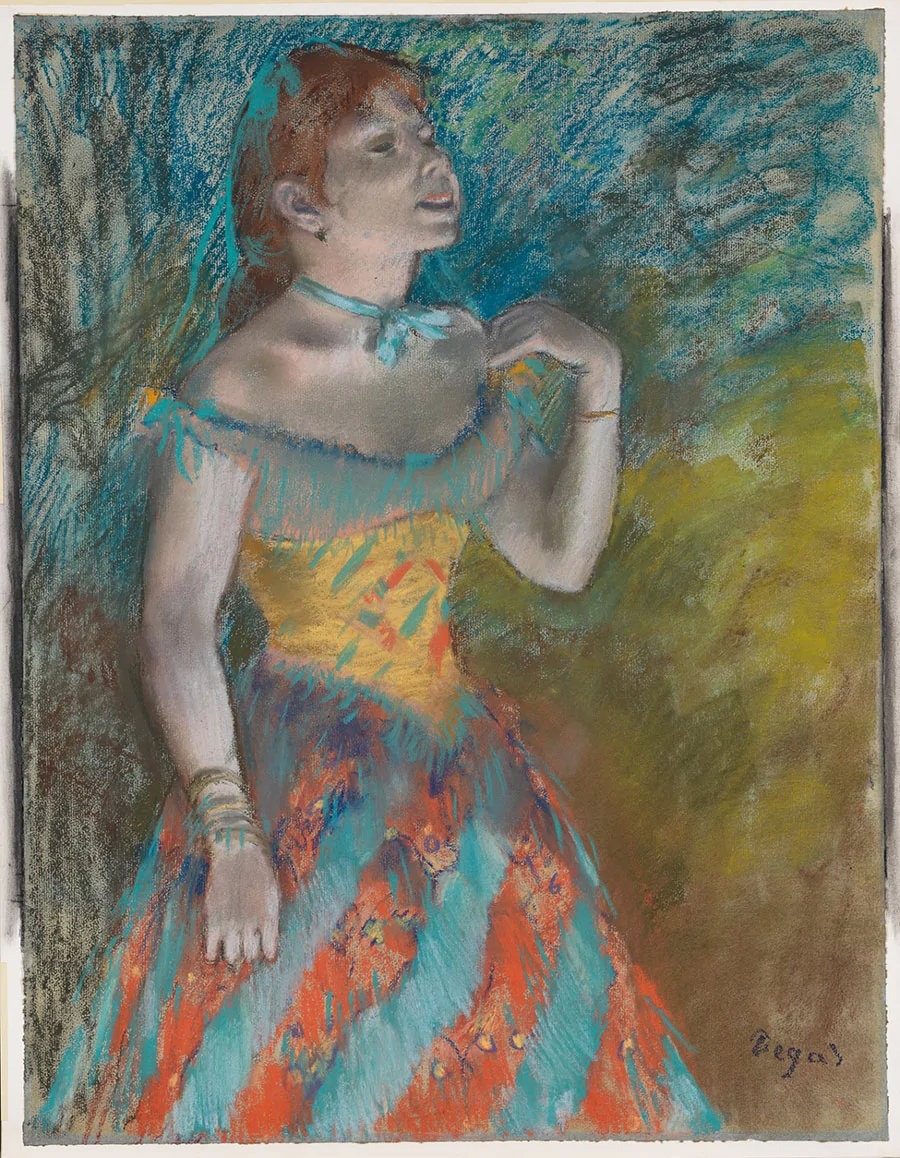
Edgar Degas, *The Singer in Green*, 1884, Pastel on light blue laid paper. 60.3 × 46.4 cm (23.75 × 18.25 in). Metropolitan Museum of Art, New York, New York, USA.

Respiratory pathogens such as influenza viruses, measles virus, *Mycobacterium tuberculosis*, and SARS-CoV-2 spread through infectious respiratory particles, such as droplets and aerosols (*1*,*2*). Singing is but one of myriad group activities in which we commonly share airspace; examples of similar such activities include performing in or attending orchestral performances, being a student in the nonvirtual classroom environment, and attending theater, operatic performances, religious or political gatherings, or sporting events. In Degas’ time, public singers had the added challenge of filling large theaters and opera houses with their voices in the absence of electronic amplification. Such vocal demands, combined with performances in often poorly ventilated indoor spaces, would have increased the risks of airborne disease transmission.

In the United States, singing in a group setting has been identified as an activity that could promote transmission risk for respiratory pathogens in investigations of tuberculosis outbreaks in a school choir in 1993–94 (*3*) and in a church choir in 1998 (*4*). In such settings, prolonged exposure and forceful vocalization create ideal conditions for aerosol spread (*5*). In 2008, investigation in a similar setting identified multiple tuberculosis cases linked to a single source, underscoring the roles of close contact and inadequate ventilation (*6*). Early in the COVID-19 pandemic, a well-documented outbreak occurred at a choir rehearsal in Skagit Valley, Washington, USA, in 2020; in that outbreak, 52 of 61 choir participants became infected with SARS-CoV-2, and 2 of them died (*7*). All of these events have supported the hypothesis of transmission risk for respiratory pathogens in association with choral singing. Elevated risk for airborne infectious disease transmission caused by aerosols has also been reported in association with playing wind instruments (*8*), coughing, talking (*9*), and other activities in which prolonged exposures can occur in poorly ventilated and crowded environments.

In response to mounting evidence during the COVID-19 pandemic, many music ensembles, including choral groups, suspended or substantially altered practices to prevent disease transmission and outbreaks. Mitigation strategies included moving rehearsals outdoors, increasing physical distancing, shortening rehearsal times, improving ventilation, using face masks, or holding virtual rehearsals and performances to maintain musical engagement while minimizing health risks (*10*–*12*). *Les Choristes* vividly portrays the setting of an age-old challenge with which society grappled in the recent pandemic setting, namely, allowing for enjoyment of close or cloistered activities without increasing respiratory disease risk, especially during epidemics or pandemics.
